# Functional benefits of the double flap technique after proximal gastrectomy for gastric cancer

**DOI:** 10.1186/s12893-021-01390-1

**Published:** 2021-11-05

**Authors:** Zenichiro Saze, Koji Kase, Hiroshi Nakano, Naoto Yamauchi, Akinao Kaneta, Yohei Watanabe, Hiroyuki Hanayama, Suguru Hayase, Tomoyuki Momma, Koji Kono

**Affiliations:** Department of Gastrointestinal Tract Surgery, Fukushima Medical University, Hikarigaoka, Fukushima-shi, Fukushima, 960-1295 Japan

**Keywords:** Gastrointestinal, Laparoscopy, Nutrition, Stomach, Oncology

## Abstract

**Background:**

Proximal gastrectomy is a widely performed procedure that has become more common with an increasing number of proximal gastric cancer cases. Several types of reconstructive procedures after proximal gastrectomy have been developed, and it remains controversial which procedure is the most advantageous with regard to the preservation of postoperative gastric stump function and nutritional status. In the present study, we retrospectively analyzed reconstructive procedures in a consecutive case series for proximal gastrectomy, primarily focusing on postoperative body weight maintenance, nutritional status, and gastric remnant functional preservation.

**Methods:**

We enrolled 69 patients who had undergone proximal gastrectomy for gastric cancer in our institute between 2005 and 2020. Short-term complications, preservation of gastric remnant functions, nutritional status, and post-operative weight changes were compared.

**Results:**

After proximal gastrectomy, the numbers of patients who underwent direct esophago-gastrostomy, jejunal interposition, double tract reconstruction, and the double flap technique were 9, 10, 14, and 36, respectively. The patients in whom the double flap technique was performed suffered no reflux esophagitis after surgery. Prevalence of gastric residual at 12 months after surgery was lowest in the double flap technique group. Moreover, the double flap technique group had a better tendency regarding post-operative changes of serum albumin ratios. Furthermore, the post-operative body weight change ratio of the double flap technique group was smallest among all groups and was significantly better than that of the double tract group.

**Conclusions:**

The double flap technique after proximal gastrectomy was considered the most effective technique for reconstruction which leads to better bodyweight maintenance, and results in less reflux esophagitis.

## Background

Even though the number of patients with gastric cancer has been decreasing, the incidence of proximal gastric cancer has been increasing worldwide [1–6]. Total gastrectomy is typically performed for most of cases of gastric cancer located in upper third of the stomach [7–11], since most of such cases are already at an advanced stage when detected, and therefore have poor prognosis [10, 12]. However, early stage proximal gastric cancer has been increasingly reported recent years in Japan and South Korea [13, 14], and it has been reported that prognosis after proximal gastrectomy is oncologically similar to that of total gastrectomy [15, 16], and it was shown that proximal gastrectomy with supra-pancreatic lymph node dissection has a favorable long-term outcome in Japan [13]. As a result, the number of patients undergoing proximal gastrectomy has been increasing in Japan. In addition, the nutritional benefits of proximal gastrectomy compared with that of total gastrectomy were recently reported [17–20]. However, in terms of quality of life(QOL) after surgery, it has been reported that patients who undergo proximal gastrectomy frequently experience regurgitation and heart burn, thus impairing their QOL [21–23]. To improve this problem, several types of reconstruction procedures after proximal gastrectomy have been developed to prevent regurgitation, such as jejunal interposition [24–26], jejunal pouch reconstruction [27, 28], gastric tube reconstruction [29], esophago-gastrostomy with fundoplication [23, 30], double tract reconstruction [18–20, 31], and double flap technique [32–34]. Particularly, the double flap technique was first introduced in 2001 by Kamikawa et al. as a reconstructive procedure which is significantly effective in preventing regurgitation after proximal gastrectomy [32]. This technique is an esophago-gastrostomy procedure, which can prevent regurgitation by developing “new cardia” because of buried esophagus in the anterior gastric wall by sero-muscular flaps. However, it is unclear whether the double flap technique has advantages in terms of postoperative nutritional status and postoperative gastric remnant functional preservation as long-term effects. Therefore, in the current study, we retrospectively conducted a comparative analysis of reconstructive procedures after proximal gastrectomy for gastric cancer.

## Methods

### Patients

Patients with gastric cancers who had undergone proximal gastrectomy between January 2005 and June 2020 at the Department of Gastrointestinal Tract Surgery, Fukushima Medical University Hospital, were enrolled in the study. All patients were preoperatively diagnosed as having gastric cancer at our institution. In all cases, the tumors were located in upper third of the stomach, and it was suggested that at least one-half of the stomach could be preserved preoperatively. Patients who underwent proximal gastrectomy with lower esophageal resection and intra-mediastinal anastomosis were excluded. The clinical and pathological data were retrospectively collected from medical records, with the last follow-up conducted in Aug 2021. These data included age, gender, body weight, hematological examination, tumor location, tumor depth, lymph nodes metastasis, and TNM classification (8th edition). Treatment was performed after obtaining written informed consent.

### Surgical procedures of proximal gastrectomy and reconstructive procedures

Proximal gastrectomy was performed under open abdominal surgery, hand assisted laparoscopic surgery (HALS), laparoscopic surgery, or robotic assisted laparoscopic surgery. D1 or D1 + lymph node dissection according to the Japanese gastric cancer treatment guidelines 2018 (5th edition) [35] was performed. Reconstruction was performed via direct esophago-gastrostomy (DEG), jejunal interposition (JIP), double tract reconstruction (DTR), or double flap technique (DFT) at the physician’s discretion. Each reconstruction procedure is summarized as follows. DEG: Esophago-gastrostomy was performed using a circular stapler inserted from a small incision in the anterior wall in the gastric remnant. The incision was then closed using absorbable sutures. Fundoplication was performed in some cases. JIP: The divided jejunum was brought up via the retro-colic route and anastomosed side-to-end with the esophagus using a circular stapler, then anastomosed end-to-side with the remaining stomach by hand-stitch. DTR: Esophago-jejunostomy and jejuno-gastrostomy were anastomosed side-by-side using a linear stapler, then the entry hole of the stapler was closed using sutures. DFT: Gastric sero-muscular flaps were prepared extra-corporeally. Then, posterior wall of full-thickness esophagus and gastric mucosa were sewn together using running sutures, and the anterior walls were sewn by layer to layer running suture or Gambee’s interrupted suture. Finally, esophago-gastrostomy was wrapped with bilateral gastric sero-muscular flaps.

### Evaluation method

After surgery, the patients were followed up at our outpatient clinic every 3 months for the first and second years after surgery, and every 6 months for a further three years. Albumin, hemoglobin, and lymphocyte count at the 3, 6, and 12 month follow-ups were evaluated. Body weight was evaluated until the 36 month follow-ups. Endoscopic examination was performed annually; however, when anastomotic stenosis was suspected, an endoscopic examination was performed and bougienage therapy was performed if necessary.

### Statistical analysis

Data were analyzed using the SPSS statistical software program version 27.0 for Mac (SPSS Inc., Chicago, Ill., USA). Continuous variables were analyzed using Student’s *t* test (2-sided test). The *χ*^2^ test with the Yates’ correction for 2 $\times$ 2 tables were used to compare categorized data. The one-way analysis of variance (ANOVA) was used to determine whether there were any statistically significant differences between the means of three or more independent groups, and the Bonferroni correction was used for post-hoc analysis. In addition, multivariate binary logistic regression analysis with corresponding odds ratios (OR) and 95% confidence intervals (CI) was performed to identify independent risk factors for a post-operative weight loss rate of > 12% at the 12 month follow-up. In this study, we set the cut-off value for postoperative body weight loss at 12%, since it is reported that patients with postoperative body weight loss > 12% have significantly poorer disease-free survivals than patients with body weight loss of less than 12% [36]. Values of p < 0.05 were considered statistically significant.

## Results

### Patient demographics

Sixty-nine patients who underwent proximal gastrectomy were enrolled. The mean age of the patients was 70.0 (range 43–94) years old, and there were 53 men (76.8%). Fourteen patients had advanced cancer (pT1a, 11; pT1b, 44; pT2, 9; pT3, 3; and pT4a, 2), although all cases were pre-operatively diagnosed as early gastric cancer. Nodal metastases were observed in five patients (N1, 4; and N2, 1). No distant metastasis was observed. All operations were undertaken with curative intent. Nineteen patients underwent open proximal gastrectomy, one patient underwent hand-assisted laparoscopic proximal gastrectomy, 36 patients had laparoscopic proximal gastrectomy, and 13 cases had robot-assisted proximal gastrectomy. Decisions on which approach was taken depended on the physician’s discretion. The patient characteristics are shown in Table 1, and there were no statistically significant differences among the groups preoperatively.

**Table.1:** Preoperative patient characteristics

	DEG (N = 9)	JIP (N = 10)	DTR (N = 14)	DFT (N = 36)	p-value
Age, y, median (median, range)	74.33, 64–94	71.5, 58–81	72.0, 43–83	70.1, 56–82	0.196
Gender (%)					0.473
Male	8 (88.9%)	8 (80.0%)	12 (85.7%)	25 (69.4%)	
Female	1 (11.1%)	2 (20.0%)	2 (14.3%)	11 (30.6%)	
BMI^a^, kg/m2 (median, range)	22.3, 20.5–32.3	21.7, 19.3–28.8	22.5, 19.0–31.9	23.1, 17.3–29.7	0.764
Albumin, g/dl (median, range)	4.0, 3.3–4.6	3.9, 3.4–4.6	4.4, 3.6–4.8	4.1, 3.0–4.6	0.084
Hemoglobin, g/dl (median, range)	13.2, 10.7–14.6	12.8, 11.7–14.9	13.7, 9.2–15.3	13.2, 8.6–15.5	0.852
Lymphocytes count, × 10^2^ /μL (median, range)	15.5, 11.8–29.1	16.1, 14.1–22.7	15.6, 11.5–31.2	15.2, 2.5–23.8	0.235

There were no statistically significant differences among the groups

^a^Body Mass Index

### Surgical background and post-operative course

Table 2 shows the surgical characteristics and post-operative courses of each reconstruction. The frequency of D1 + lymph node dissection in DFT group was significantly higher than those in the other groups. Most cases of DEG and JIP were performed via open laparotomy; in contrast, all cases of DTR were performed via laparoscopic surgery, and robotic surgery was only performed in the DFT group. Pathological depth of tumor invasion, lymph node metastasis, and pathological stage were not significantly different between the groups. The hospital stay length of the DFT group was the shortest, and significantly shorter than those of the DEG and JIP groups (p < 0.001, p = 0.014, data not shown in Table 2). Post-operative short-term complications including anastomotic leakage and pancreatic fistula did not significantly differ among the groups. No surgical death was observed in any of the groups. Comparison of long-term complications revealed no significant differences among the groups regarding the rate of anastomotic stenosis, as shown in Table 2. Although ratios of suffering reflux gastritis above grade A in Los Angeles classification after surgery between each reconstruction group were not significantly different, only DFT cases had no reflux esophagitis and the ratio of the administration of proton pump inhibitors of DFT was only 16.7%. Moreover, the DFT group had the lowest rate of gastric residual according to observations made using a post-operative upper gastrointestinal endoscope.

**Table.2:** Operative results and post-operative courses

	DEG (N = 9)	JIP (N = 10)	DTR (N = 14)	DFT (N = 36)	p-value
Extent of dissection (%)					
D1	5 (55.6%)	4 (40.0%)	4 (28.6%)	3 (8.3%)	< 0.001*
D1 +	4 (44.4%)	6 (60.0%)	10 (71.4%)	33 (91.7%)	
Approach					
Open laparotomy	6 (66.7%)	10 (100%)	0 (0%)	3 (8.3%)	< 0.001*
HALS	1 (11.1%)	0 (0%)	0 (0%)	0 (0%)	
Laparoscopy	2 (22.2%)	0 (0%)	14 (100%)	20 (55.6%)	
Robotic laparoscopy	0 (0%)	0 (0%)	0 (0%)	13 (36.1%)	
Depth of invasion (%)					
T1a	2 (22.2%)	0 (0%)	4 (28.6%)	5 (13.9%)	0.446
T1b	5 (55.6%)	9 (90.0%)	8 (57.1%)	22 (61.1%)	
T2	0 (0%)	1 (10.0%)	1 (7.1%)	7 (19.4%)	
T3	1(11.1%)	0 (0%)	1 (7.1%)	1 (2.8%)	
T4a	1 (11.1%)	0 (0%)	0 (0%)	1 (2.8%)	
Lymph node metastasis					
N0	9 (100.0%)	10 (100.0%)	12 (85.7%)	33 (91.7%)	0.462
N1	0 (0%)	0 (0%)	1 (7.1%)	3 (8.3%)	
N2	0 (0%)	0 (0%)	1 (7.1%)	0 (0%)	
Pathological Stage(UICC 7th)					
IA	7 (77.8%)	9 (90.0%)	11(78.6%)	26 (72.2%)	0.590
IB	0 (0%)	1 (10.0%)	1 (7.1%)	8 (22.2%)	
IIA	1 (11.1%)	0 (0%)	1 (7.1%)	1 (2.8%)	
IIB	1 (11.1%)	0 (0%)	1 (7.1%)	1 (2.8%)	
Hospital stay (days, mean ± SD)	18.1 ± 5.8	15.2 ± 5.6	12.2 ± 3.6	10.5 ± 3.3	< 0.001*
Short-term complications					
CD^a^ grade II					
Ileus	0 (0%)	0 (0%)	1 (7.1%)	3 (8.3%)	0.650
Pneumonia	0 (0%)	0 (0%)	1 (7.1%)	0 (0%)	0.263
Postoperative hemorrhage (%)	1 (11.1%)	1 (10%)	0 (0%)	1 (2.8%)	0.456
Anastomotic leakage	0 (0%)	0 (0%)	0 (0%)	1 (2.8%)	0.818
Pancreatic fistula	0 (0%)	0 (0%)	1 (7.1%)	1 (2.8%)	0.690
Long-term complications					
Anastomotic stenosis^b^	1 (11.1%)	1 (10%)	3 (21.4%)	3 (8.3%)	0.935
Reflux esophagitis^c^	2 (22.2%)	1 (10%)	3 (21.4%)	0 (0%)	0.203
Gastric residual^d^	3 (33.3%)	7 (70%)	2 (14.3%)	5 (13.9%)	0.017*
PPI^e^ administration	8 (88.9%)	5 (50%)	2 (14.3%)	6 (16.7%)	< 0.001

Hospital stay in the DFT group was the shortest, and was significantly shorter than those in the DEG and JIP groups. A comparison of long-term complications showed no significant differences regarding rate of anastomotic stenosis. Reflux esophagitis did not occur in the DFT group, which also had the least frequent gastric residual

^a^Clavien–Dindo classification

^b^Anastomotic stenosis which required balloon dilatation

^c^Reflux esophagitis grade ≥ A in Los Angeles classification

^d^Gastric residual observed by endoscopy at 1 year after surgery

^e^Post-operative proton pump inhibitor administration

^*^Statistically significant

### Indicators of post-operative nutritional status changes

Table 3 shows post-operative indicators of nutritional status changes. Changes in serum albumin ratios at the 6- and 12-month follow-ups showed significant differences among the reconstructive groups, and the DFT group had better tendency in serum albumin ratio. Hemoglobin ratios and lymphocyte count ratios did not significantly differ among the groups.

**Table.3:** Post-operative indicators of nutritional status changes

	Period	DEG (N = 9)	JIP (N = 10)	DTR (N = 14)	DFT (N = 36)	p-value
Albumin change	3 mths	89.5 ± 8.1	98.0 ± 10.4	92.7 ± 6.7	97.2 ± 9.4	0.134
	6 mths	89 ± 9.9	103.1 ± 10.2	93.9 ± 11.0	100.1 ± 11.6	0.041*
	12 mths	94.2 ± 5.5	106.8 ± 12.2	97.4 ± 5.3	100.2 ± 9.1	0.037*
Hemoglobin change	3 mths	94.3 ± 6.4	90.7 ± 9.2	91.43 ± 6.6	103.9 ± 48.9	0.655
	6 mths	88.4 ± 3.2	89.7 ± 10.6	92.7 ± 6.2	105.5 ± 54.3	0.636
	12 mths	94.8 ± 7.0	92.4 ± 8.6	92.5 ± 5.8	98.2 ± 9.5	0.172
Lymphocyte count change	3 mths	69.6 ± 14.0	88.5 ± 28.6	87 ± 28.3	118.1 ± 51.0	0.107
	6 mths	61.6 ± 39.1	79.8 ± 25.4	90.8 ± 26.1	121.8 ± 69.6	0.183
	12 mths	67.2 ± 4.6	100.8 ± 48.1	103 ± 39.0	125.2 ± 88.9	0.583

Values are percentage of post-operative to the pre-operative (mean ± SD)

Changes in serum albumin ratios showed that the DFT group has better tendency in serum albumin ratios. Hemoglobin ratios, and lymphocyte count ratios were not significantly different among the groups

*Statistically significant

### Post-operative body weight changes

**Fig. 1:**
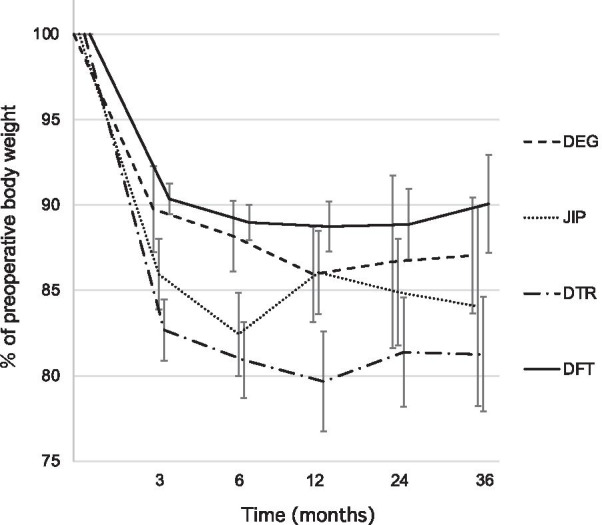
The percentage of post-operative body weight to the pre-operative. Data are expressed as mean ± standard error. The body weight loss ratio of the DFT group was significantly better than that of the DTR group at the 3, 6, and 12 month follow ups (p = 0.001, 0.003, and 0.013, respectively)

Figure 1 shows body weight changes over the first 36 months following proximal gastrectomy in each reconstruction group. The one-way ANOVA detected significant differences among the groups at the 3, 6, and 12 month follow-ups (p = 0.001, 0.002, and 0.022, respectively) and the DFT group had the most favorable results. In addition, multiple comparisons showed that the body weight loss ratio in the DFT group was significantly better than that in the DTR group at 3, 6, and 12 month follow-ups (p = 0.001, 0.003, and 0.013, respectively). Furthermore, multivariate analysis revealed that performing a reconstruction method other than DFT was an independent risk factor of a post-operative weight loss rate of > 12% at the 12 month follow-up (Table 4).

**Table.4:** Multivariate analysis for risk of post-operative weight loss rate above 12% at 1-year after surgery

Variables	OR	95% CI	p value
Age < 70	0.536	0.148–1.943	0.343
Male	1.884	0.376–9.452	0.441
Pre-operative BMI^a^ < 22	2.925	0.724–11.823	0.132
Laparotomy and HALS^b^	4.319	0.708–26.355	0.113
D1 + Lymph node dissection	2.85	0.456–17.816	0.263
Short-term complication^c^	4.484	0.274–73.37	0.293
Reconstruction with other than DFT^d^	6.037	1.226–29.732	0.027*

Multivariate analysis revealed that using a reconstruction method other than DFT was an independent risk factor for a post-operative weight loss rate of > 12% at 12 months after surgery

^a^Body Mass Index

^b^Hand Assisted Laparoscopic Surgery

^c^Complications grade II or above in Clavien–Dindo classification

^d^Double Flap Technique

^*^Statistically significant

## Discussion

In the present study, we retrospectively analyzed our consecutive case series for proximal gastrectomy, with particular focus on postoperative body weight maintenance, nutritional status, and gastric remnant functional preservation. We found that DFT is the most effective reconstructive procedure to prevent reflux esophagitis, since reflux esophagitis did not occur in any patients in the DFT group, whereas it did occur in the other groups. Moreover, we showed that the rate of anastomotic stenosis after DFT (8.3%) was less frequent in comparison to other reconstruction procedures. However, care should be taken when interpreting the anastomotic stenosis date. It is generally accepted that a circular stapler is widely used for esophago-jejunostomy [37–39], and the stricture rate of stapler anastomosis has been reported to be high compared to that of hand-sewn anastomosis [40–42]. Since esophago-gastrostomy in DFT is performed by hand-sewn sutures, it makes anastomosis soft and flexible, and can prevents anastomotic stenosis [43]. In other words, the low anastomotic stenosis rate in DFT may be due to hand-sewn anastomosis, not the DFT procedure itself. The rate of anastomotic stenosis after DFT has been reported to be 5.5–9% [34, 43, 44], and was 8.3% in the present study. Therefore, we still need to improve and modify the DFT procedure to the point where it can prevent anastomotic stenosis more completely. At our institution, we currently employ Gambee’s method for suturing the anterior wall of esophago-gastrostomy in the DFT reconstruction, instead of using a layer-to-layer running suture. Moreover, there are some reports that DFT was performed via laparoscopic surgery, which may be more beneficial to the patient because it is a minimally invasive procedure [17, 34]. However, laparoscopic DFT is cumbersome due to restriction of movement; surgeons therefore need to be particularly skilled in laparoscopic suturing. However, this issue may be resolved by robotic surgery [45]. In the present study, robotic-assisted DFT was performed in 13 cases, with favorable results.

In the current study, we showed that employing the double flap technique for reconstruction after proximal gastrectomy has the most favorable outcome with regard to post-operative body weight loss. We believe that one possible reason for it is that regurgitation occurs less frequently, possibly leading to better food intake. On the other hand, DTR is the worst of body weight loss. DTR contains Roux-en Y reconstruction as part of it, ingested food may solely pass through the jejunum and small amount of food may enter the gastric remnant, possibly resulting in the worst body weight loss. Unfortunately, we were not able to show a solid advantage of DFT in postoperative nutritional status within hematological examination compared to other reconstructive procedures, although there was minor advantage in DFT group for albumin change.

Although the present study has provided some important information for clinical practice, it has some limitations. In particular, this was a retrospective study with a relatively small sample size from a single institution. Further accumulation of cases is required. Second, the study may have biases. We did not evaluate the size of the gastric remnant, which may affect post-operative body weight loss and nutritional status. Other biases are the follows. This study contains long period of cases. DFT were performed in recent years and various surgical outcomes can be affected by progresses in surgical devices or techniques like laparoscopic surgery and robotic surgery. In addition, reconstruction was selected by physician’s choice, which may be another potential bias.

## Conclusions

We here demonstrated the advantages of DFT after proximal gastrectomy for gastric cancer. DFT markedly decreased the risk of post-operative body weight loss and reflux esophagitis in comparison with other reconstructive procedures for proximal gastrectomy.

## Data Availability

The datasets used and/or analyzed during the current study are available from the corresponding author on reasonable request.

## Abbreviations

HALS: Hand assisted laparoscopic surgery
DEG: Direct esophago-gastrostomy
JIP: Jejunal interposition
DTR: Double tract reconstruction
DFT: Double flap technique
ANOVA: Analysis of variance
OR: Odds ratios
QOL: Quality of life
